# Supplementary material for: Minigene-Based Splice Assays Reveal the Effect of Non-Canonical Splice Site Variants in USH2A
Source: Int J Mol Sci. 2022 Nov 1;23(21):13343. doi: 10.3390/ijms232113343 (PMC9654511; doi:10.3390/ijms232113343)
Supplement: Supplementary file 1 [file ijms-23-13343-s001.zip › ijms-1923533-supplementary.pdf]

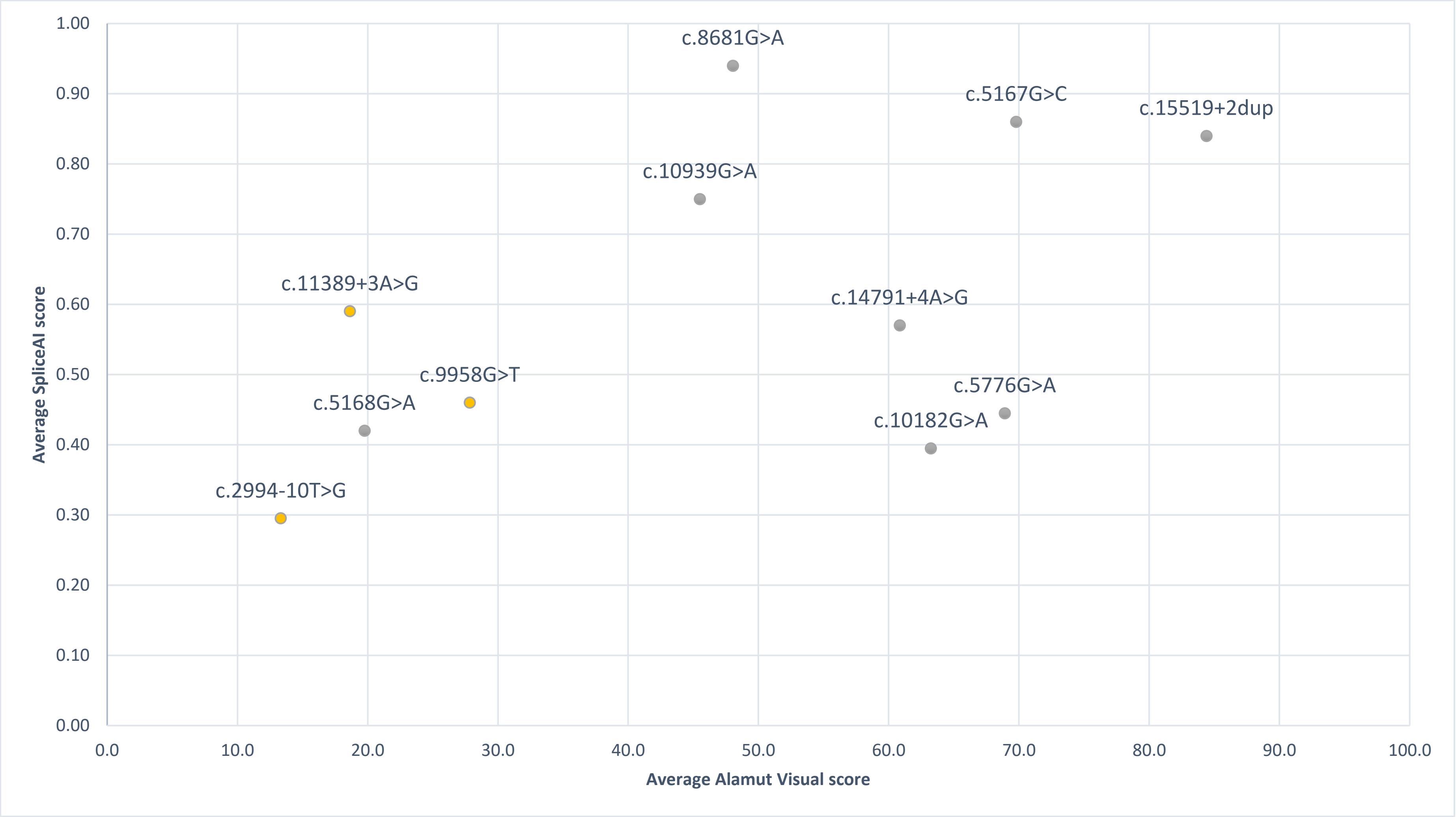

Figure S1. Scatter plot of the average Alamut Visual splice prediction scores compared to the average of the two highest SpliceAI prediction scores. Three variants (c. 2994-10T>G, c.9958G>T and c.11389+3A>G) with remaining conventionally splice RNA are depicted in orange.

Table S1. SpliceAI prediction scores.

NCSS variant	Average Alamut Visual prediction (%)	Average SpliceAI score*	SpliceAI acceptor gain	SpliceAI acceptor loss	SpliceAI donor gain	SpliceAI donor loss	SpliceAI acceptor gain; delta position	SpliceAI acceptor loss; delta position	SpliceAI donor gain; delta position	SpliceAI donor loss; delta position
c.2994-10T>G	13.3	0.30	0.01	0.30	0.00	0.29	-39	-10	61	-173
c.5167G>C	69.8	0.86	0.00	0.77	0.00	0.95	390	179	-45	0
c.5168G>A	19.7	0.42	0.01	0.39	0.00	0.45	-2	0	1291	-130
c.5776G>A	68.9	0.45	0.00	0.49	0.10	0.40	1840	203	-11	0
c.8681G>A	48.0	0.94	0.00	0.90	0.00	0.98	621	122	-87	0
c.9958G>T	27.8	0.46	0.07	0.14	0.29	0.63	1068	-1140	61	0
c.10182G>A	63.2	0.40	0.00	0.39	0.00	0.40	-185	223	-860	0
c.10939G>A	45.5	0.75	0.05	0.69	0.02	0.81	720	198	58	0
c.11389+3A>G	18.6	0.59	0.00	0.56	0.07	0.62	701	160	46	3
c.14791+4A>G	60.8	0.57	0.00	0.45	0.00	0.69	380	212	53	4
c.15519+2dup	84.4	0.84	0.00	0.72	0.21	0.96	815	223	-121	2

* For the average SpliceAI prediction, only the two highest scores were considered. NCSS: Non-canonical splice site

Table S2. Sequences of primers used to generate constructs for the minigene splice assays.

NCSS variant	Forward primer	Reverse primer	Insert size (kb)
c.2994-10T>G	GGGGACAAGTTTGTACAAAAAAGCAGGCTTCtgctcaaaacattgcgtgc	GGGGACCACTTTGTACAAGAAAGCTGGGTGtgtccagggaagaatcattagg	3.4
c.5167G>C and c.5168G>A	GGGGACAAGTTTGTACAAAAAAGCAGGCTTCcccaagtctctcccaggaag	GGGGACCACTTTGTACAAGAAAGCTGGGTGagccaacaatacacaagaggac	3.7
c.5776G>A	GGGGACAAGTTTGTACAAAAAAGCAGGCTTCtcaccaccatccctctgaag	GGGGACCACTTTGTACAAGAAAGCTGGGTGcaggccacaagatcgagttg	3.6
c.8681G>A	GGGGACAAGTTTGTACAAAAAAGCAGGCTTCcaactagcatgtggtgagagc	GGGGACCACTTTGTACAAGAAAGCTGGGTGcactatgtgctggtatgtgcc	3.8
c.9958G>T	GGGGACAAGTTTGTACAAAAAAGCAGGCTTCaagaagaggaggagcaggac	GGGGACCACTTTGTACAAGAAAGCTGGGTGgtcactcaagagctctgatgg	2.6
c.10182G>A	GGGGACAAGTTTGTACAAAAAAGCAGGCTTCaaacaattcaggaccccagg	GGGGACCACTTTGTACAAGAAAGCTGGGTGgcttgaagtgcatttggagc	6
c.10939G>A	GGGGACAAGTTTGTACAAAAAAGCAGGCTTCtgtggatgatgagcactggg	GGGGACCACTTTGTACAAGAAAGCTGGGTGgaaggaggtggctgacaatg	2.4
c.11389+3A>G	GGGGACAAGTTTGTACAAAAAAGCAGGCTTCaggccaagatttcgacatgg	GGGGACCACTTTGTACAAGAAAGCTGGGTGctaccgatggctcaaatggc	3.4
c.14791+4A>G	GGGGACAAGTTTGTACAAAAAAGCAGGCTTCccagcacgatgaagactcttg	GGGGACCACTTTGTACAAGAAAGCTGGGTGtctgggtggaggggtataca	5.6
c.15519+2dup	GGGGACAAGTTTGTACAAAAAAGCAGGCTTCgtacctctgtgtgctgcatg	GGGGACCACTTTGTACAAGAAAGCTGGGTGtctaactctggacctcgtgatc	2.4
RHO exons 3 to 5 (RT-PCR)	cggaggtcaacaacgagtct	aggtgtaggggatgggagac	na
GAPDH control (RT-PCR)	ctgcaccaccaactgcttag	agctcagggatgaccttgc	na

Sequence of the Gateway® attB site is depicted in upper case, the gene-specific sequences in lower case letters. kb: kilobase, na: not applicable, NCSS: Non-canonical splice site
